# No need to wait for the blood tests: the clinical diagnosis of hypocalcemia

**DOI:** 10.11604/pamj.2014.17.65.3881

**Published:** 2014-01-27

**Authors:** Theocharis Koufakis, Ioannis Gabranis

**Affiliations:** 1 Department of Internal Medicine, General Hospital of Larissa, Larissa, Greece

**Keywords:** Hypocalcemia, Trousseau sign, ECG, QT interval

## Image in medicine

A 75 years old woman, with a history of cervical cancer, liver and pulmonary metastases, presented to the Emergency Department of our hospital with generalized tonic-clonic seizures. She had an abnormal ECG, mainly characterized by a prolonged QT interval. Trousseau sign was elicited after the sphygmomanometer cuff was inflated to more than the systolic blood pressure on her left arm. In view of these findings, we strongly considered hypocalcemia as the cause of seizures and laboratory investigations confirmed our primary clinical suspicion: she had a corrected blood calcium level of 7 mg/dl (normal values 8.1-10.4 mg/dl). The patient was admitted for further evaluation and her initial treatment included intravenous administration of calcium gluconate. A cranial CT scan was performed, which excluded brain metastases. She was discharged five days later on oral calcium supplements. In conclusion, nowadays, that technology has deeply penetrated the clinical practice, physicians should never forget that in most cases, a thorough physical examination of the patient is enough to set the diagnosis.

**Figure 1:**
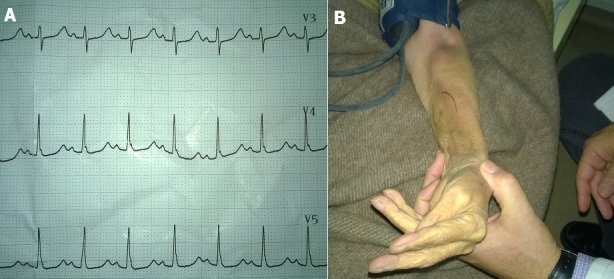
A) Prolonged QT interval on ECG and B) Positive Trousseau sign in a patient with hypocalcemia

